# Prevalence and spatial distribution of bovine brucellosis in San Luis and La Pampa, Argentina

**DOI:** 10.1186/s12917-015-0535-1

**Published:** 2015-08-15

**Authors:** M. N. Aznar, F. J. Linares, B. Cosentino, A. Sago, L. La Sala, E. León, S. Duffy, A. Perez

**Affiliations:** Área de Patología, Epidemiología y Medicina Preventiva. Instituto de Patobiología. CICVyA, Instituto Nacional de Tecnología Agropecuaria, Hurlingham, PC 1688 Argentina; Faculty of Veterinary Medicine, Department of Infectious and Parasitic Diseases, Research Unit of Epidemiology and Risk Analysis Applied to Veterinary Sciences (UREAR), University of Liege, Liege, PC 4000 Belgium; Dirección de Epidemiología y Análisis de Riesgo, Dirección Nacional de Sanidad Animal, Servicio Nacional de Sanidad y Calidad Agroalimentaria, Ciudad Autónoma de, Buenos Aires, PC 1063 Argentina; Centro Regional La Pampa-San Luis, Servicio Nacional de Sanidad y Calidad Agroalimentaria, La Pampa, PC 6300 Argentina; Cátedra de Parasitología Cínica. Departamento de Biología, Bioquímica y Farmacia, Universidad Nacional del Sur, Bahía Blanca, PC 8000 Argentina; Centro de Estudios Cuantitativos en Sanidad Animal, Facultad de Ciencias Veterinarias. Universidad Nacional de Rosario, Casilda, PC 2170 Argentina; Department of Veterinary Population Medicine, College of Veterinary Medicine. University of Minnesota, Minnessota, MN 55113 USA

**Keywords:** Bovine brucellosis, Prevalence, Spatial analysis, Argentina

## Abstract

**Background:**

Bovine brucellosis (BB) is a zoonotic disease caused by *Brucella abortus.* BB is endemic in Argentina, where vaccination with *Brucella abortus* strain 19 is compulsory for 3-to-8 month-old heifers. The objectives of this study were to quantify the prevalence of BB and to identify factors associated with its occurrence, along with the spatial distribution of the disease, in the provinces of La Pampa and San Luis. A two-stage random sampling design was used to sample 8,965 cows (3,513 in La Pampa and 5,452 in San Luis) from 451 farms (187 in La Pampa and 264 in San Luis).

**Results:**

Cow and herd prevalence were 1.8 % (95 % CI: 1.3–2.2; *n* = 157) and 19.7 % (95 % CI: 17.0–22.4; *n* = 89), respectively. Both cow-level and herd-level prevalence in La Pampa (2.4 and 26.0 %, respectively) were significantly higher than in San Luis (1.4 and 15.5 %, respectively). There were not differences between the proportions of reactive cattle compared to that obtained in a survey conducted in 2005. However, herd prevalence in La Pampa was significantly (*P < 0.05*) higher compared to that study. Disease was found to be spatially clustered in west La Pampa. The lower the bovine density and the calf/cow ratio, the higher odds of belonging to the cluster.

**Conclusions:**

The increase of farm prevalence in the last five years suggests that the disease is spreading and that control measures should be applied in the region.

The cluster of infected farms was located in the west region of La Pampa. There, farms have lower animal densities and smaller cow/calf indices compared to the rest of the province. Although western La Pampa has more infected herds, within-farm prevalence was not higher, which suggests that the control program has been relatively successful in controlling the disease at the farm level, and/or that low animal density inherently results in low disease prevalence. Our results provide baseline information on the epidemiology of BB and its potential pattern of transmission in Argentina, which will ultimately help to improve BB control programs in the country.

## Background

Brucellosis is considered one of the most widespread zoonotic diseases worldwide [1]. The disease is caused by various bacteria belonging to the genus *Brucella*, which affects many mammalian species, including cattle, goats, pigs, and sheep. Bovine brucellosis (BB), which is predominantly caused by *Brucella abortus*, is usually detected in pregnant females that abort [2], which may develop life-long infection. BB reduces fertility and milk production and may be transmitted to humans by direct or indirect contact with infected animals [1]. Because prevalence in animals influences disease incidence in humans [3], control in animal populations is important to improve the productive capability of herds and to protect the human population from infection.

BB is endemic in Argentina. In 1998, the Argentine dairy industry launched an incentive program for BB-free herds, which encouraged milk producers to improve the sanitary status of their herds by reducing the prevalence [4]. Currently, the disease is more frequently controlled in dairies compared to beef farms [5], and 5,870 dairy farms (53 % of the total dairy farms) have been officially certified as BB-free (SENASA, unpublished data). The Argentine National Bovine Brucellosis Control Program [6] establishes the compulsory vaccination of 3-to-8 month-old heifers with Brucella abortus strain 19. The province of Tierra del Fuego, in southern Patagonia, is BB-free and vaccination is forbidden there [7].

Quantitative knowledge on the disease prevalence and spatial distribution is prerequisite for designing and assessing the evolution of disease control programs [8].

**Fig. 1:**
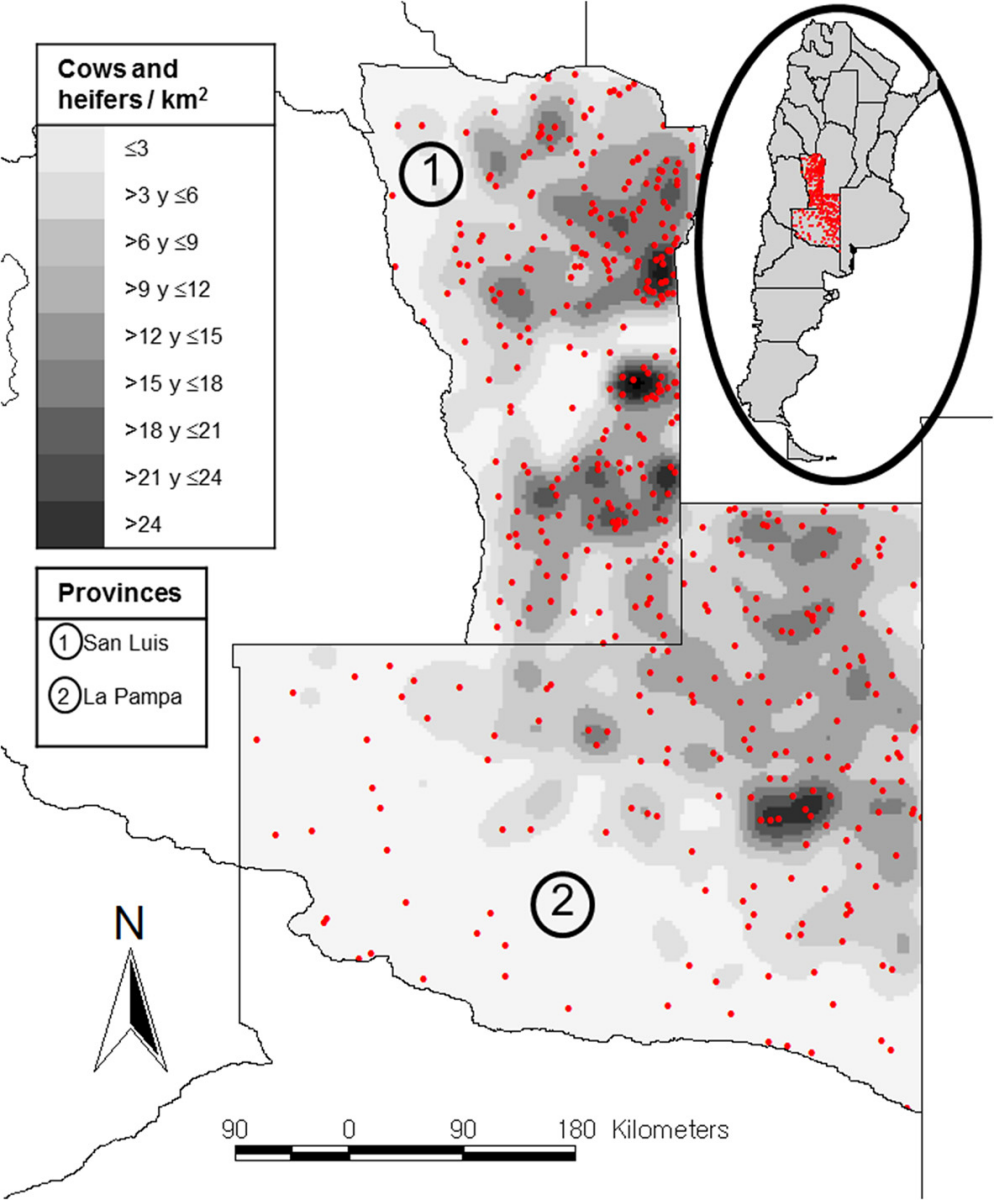
Map of Argentina (circle) showing the studied area (red dots), and a detail of La Pampa and San Luis provinces showing a kernel density of cows and heifers (tones of grey) and the geographic distribution of the 451 sampled farms (red dots)

The economic role of cattle breeding, in La Pampa and San Luis provinces (Fig. 1), is prominent, particularly for beef cattle. Approximately 29–35 % of the females and 1 % of the bulls produced in the region are shipped into premises located in other regions of Argentina (SENASA, unpublished data). Regarding La Pampa, this province includes three different productive areas referred to as northeast, central (Caldenal), and west [9]. The west region is mostly (98 %) covered with xerophytic native forest and grassland. It is an area with big extensive breeding farms and comprised mainly of cow-calf operations with a low calf/cow index. Similarly, 88 % of the central area is comprised of native forest and grassland, but includes a combination of both cow-calf and fattening operations with a higher calf/cow index. In the northeastern area, only 25 % of the land is covered by forests and grassland, and calf breeding/fattening activities are combined in the same operations or conducted separately with the highest calf/cow index.

Knowledge on the epidemiological status of BB in the region is scarce. In 2005, SENASA conducted a survey in La Pampa and San Luis provinces to estimate the prevalence of the disease in beef cattle, and reported a cow-level prevalence of 2.6 and 1.8 %, and a herd-level prevalence of 11.0 and 9.0 %, respectively (SENASA, unpublished data). However, no structured assessment of the epidemiological situation of BB in the region has been conducted since then.

The objectives of the study here were 1) to estimate the BB prevalence at farm and province levels, and 2) to identify factors associated with the occurrence of disease and their association with spatial clustering of cases. Results will help to characterize the epidemiology of the disease and, ultimately, to evaluate and improve the effectiveness of BB control programs in the region.

## Methods

La Pampa has a cattle population of 2.7 million distributed in 8,000 farms, whereas San Luis has 1.4 million head in 6,000 farms (SENASA 2013, unpublished data). A two-stage random-sampling design was used to estimate the proportion of infected cows. In the first stage, farms (primary sampling units) were selected with a probability of selection proportional to the number of cattle in the farm. In the second stage, cattle (secondary sampling units) were randomly selected from each farm.

The number of farms to be selected in the region under study (n) was calculated using the following formula:

$$ \mathrm{n}=\frac{\mathrm{p}\times \left(1-\mathrm{p}\right)\times {\mathrm{z}}^2\times \left[\mathrm{R}\mathrm{O}\mathrm{H}\times \left(\mathrm{b}-1\right)+1\right]}{{\mathrm{e}}^2\times \mathrm{b}} $$

where p is the expected proportion of cows reactive to brucellosis, z is the level of confidence, ROH is the rate of homogeneity, b is the number of animals selected per farm (may be variable: the fewer individuals per farm the greater the number of farms to be selected) and e is the acceptable absolute error. Our assumptions were: expected proportion of cows reactive to brucellosis: 2.6 % for La Pampa and 1.8 % for San Luis (based on the SENASA survey 2005), level of confidence: 95 %; relative error: 30 %; rate of homogeneity: low.

The result was that a minimum of 415 farms were needed.

A total of 19 cows was chosen per farm taking into account that it is the number for detecting the disease if its prevalence is ≥ 0.15 and that it was also a feasible number for operative reasons. So, a total of 7,885 cows was required.

In farms where the population size was <19, the entire population was sampled. In some farms more than 19 animals were sampled to compensate such reduction and reach the total number of samples targeted for the region. The software ProMesa 1.3 was used in sample size calculations [10].

Data on cattle population, farm characteristics (location and size) and cattle movements during 2009 in the region under study were obtained from the SENASA Integrated Sanitary Management System (Sistema Integrado de Gestión de Sanidad Animal - “SIGSA”) for each sampled farm.

Serum samples were obtained by SENASA local veterinarians during 2010.

### Ethical approval

The INTA CICUAE committee ruled that no formal ethics approval was required in this case, in which the sampling was part of a SENASA surveillance activity. In all cases, informed verbal consent was obtained from the cattle owners.

### Serology

The buffered plate agglutination test (BPAT) was used as screening test (sensitivity [Se] = 95.4 %; specificity [Sp] = 97.7 %). Positive samples were confirmed using the 2- mercaptoethanol (2ME; Se = 88.4 %: Sp = 91.5 %) and the serum agglutination tests (SAT; Se = 75.9 %: Sp = 95.7 %) [11], as stated by the SENASA Resolution 438/2006 [12].

Those animals that tested positive for BPAT, 2ME, and SAT tests were classified as positive, and as negative otherwise. According to the Argentine legislation [6], premises with at least one reactive animal were defined as BB-infected.

### Statistical analysis

Prevalence was computed for each province (San Luis and La Pampa). Results were compared with those obtained in the SENASA 2005 survey. Prevalence values were compared between provinces and years using the Fisher’s exact test. Statistical analyses were performed using Statistix 8.0 [13].

The association between various independent variables and outcomes (infected/not infected farm; count of reactive cows per farm; located inside/outside a high prevalence cluster) were evaluated.

A first generalized linear model (GLM; logistic) was built to assess association between the dependent variable “infected/not infected farm” and independent variables, which were categorized as dichotomous using two different cut-off values: a) the median considering only the sampled farms; b) the median considering the total beef farms population in both provinces. The list of independent variables and cut-off values are presented in Table 1. A second GLM (Poisson) was carried out for evaluating the association between the same variables and cut-off values used for the first model and the count of reactive cows per farm as dependent variable.

**Table 1:** Cut-off values for categorization of independent variables: (A) median of sampled farms; (B) median of the total beef farms population in both provinces

Independent variable	Cut-off (A)	Cut-off (B)
Farm surface (Has)	500	300
Bovine density (animals/Has)	0.5	0.25
Calf/cow ratio	0.5	0.36
Number of cows per farm	600	85
Number of heifers per farm	50	23
Number of cows and heifers shipped to and from the farm during the previous year	50	25
Number of farms shipping cattle to/from another farm during the previous year	10	15

Clustering of BB-infected farms was assessed using two methods suggested for outbreak investigations [14]. First, the Cuzick- Edwards’ test [15] was used to detect any overall, globally clustered distribution of cases at the farm level using the ClusterSeer 2.5.1 software [16]. The test statistic T*k,* which represents the number of cases (*i.e.* farms) that are nearest to each individual case, was calculated. Here, the first ten orders (*k* = 1–10) of nearest-neighbors measurements were examined and significance was computed using 999 Monte Carlo iterations. After the initial detection of a global clustering of positive farms, further analyses were done to locate and characterize the cluster/s using the spatial scan statistic [17]. A Bernoulli model was implemented to identify clusters of positive farms, followed by a Normal model to detect clustering of farms with high prevalence. Cluster analyses were done using the SatScan software [18].

Following the spatial analysis and identification/location of clusters, herds were categorized as located within/outside a cluster. Then, a third GLM was built to investigate association between the variable of interest (located inside/outside a cluster) and the independent variables with the same cut- off mentioned above (Table 1).

Model building began by a univariate analysis of each variable. Variables having a significant univariate test at an initial p-value cut-off point of 0.20 were included in a multivariate model. Confounding was considered if removing single variables from the multivariable model changed any of the parameter estimates by 20 % or more compared to the full model [19]. At the end of the process, the model contained only significant independent variables without confounders. To be included in the models, independent variables were required to be significant under both cut-off scenarios considered (sampled farms and population-level medians).

The associations between the outcome and independent variables were quantified by the estimation of the odds ratios (OR) and their 95 % confidence intervals (95 % CI), for the two cut-off levels. Final model selection was based on the Deviance Information Criterion (DIC).

The sampled farms were mapped in ArcGIS 9.3 software [20]. The spatial distribution of cows and heifers was mapped using a kernel density function [21] of the same software (Fig. 1).

## Results and discussion

In 70 % of the farms, the originally 19 targeted samples were obtained, whereas in the remaining 30 % the number of samples varied between 10 and 25, depending on the number of cows per farm. The geographic distribution of the sampled farms, overlapping the density of cows and heifers in each province (expressed as number of animals per km^2^) is shown in Fig. 1.

**Fig. 2:**
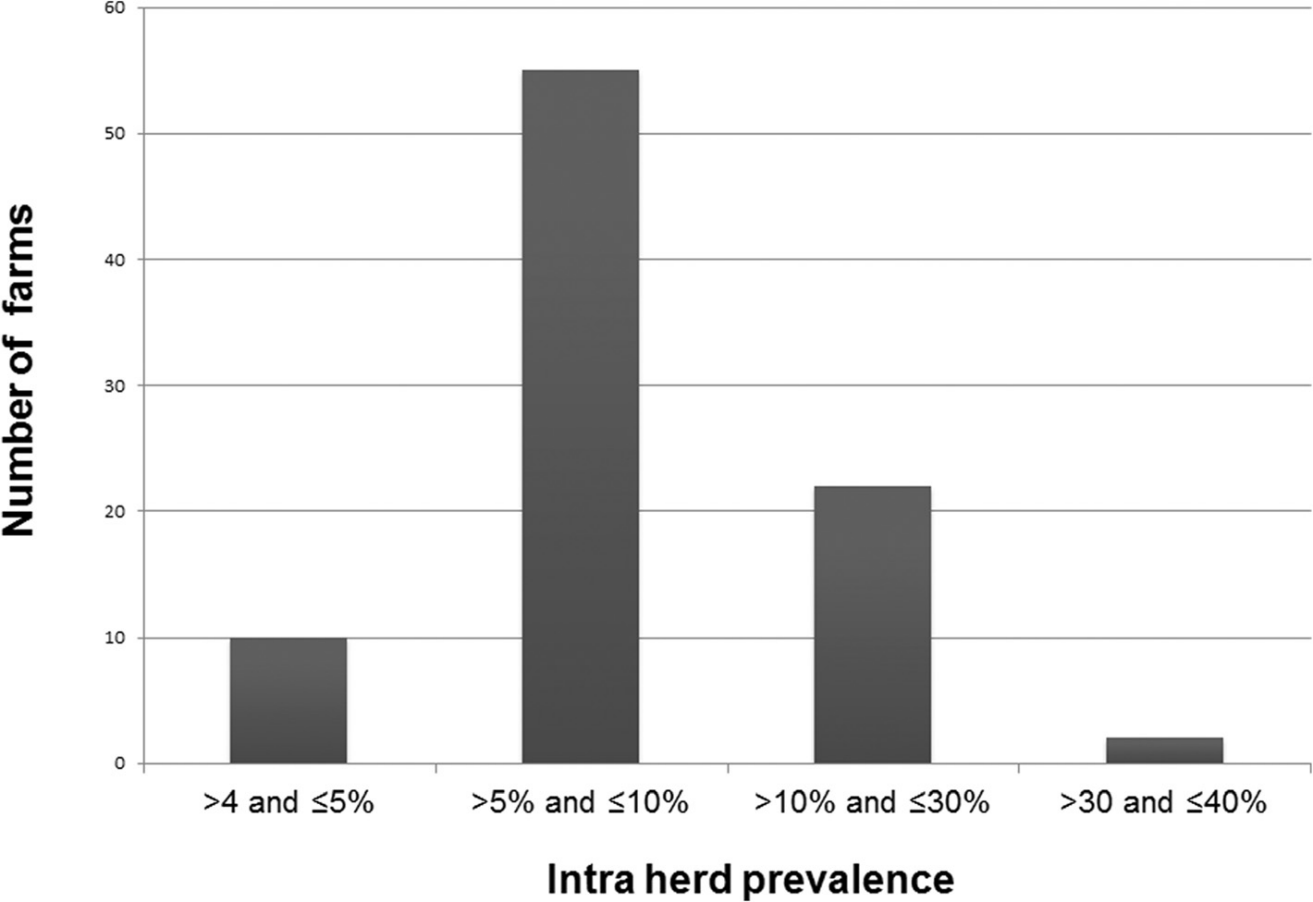
Frequency distribution of the infected farms (*n* = 89) by intra-herd prevalence

Almost 20 % of the farms were infected, showing not only a high frequency but also a high dispersion of the infection, with the majority of the infected farms having a proportion of reactive cows lower than 10 %. Intra-farm prevalence ranged from 4.3 to 40 %, with a median prevalence of 5.3 % (Fig. 2).

The prevalence of reactive cows and infected farms were higher in La Pampa than in San Luis (*P < 0.05*; Table 2). However, the observed differences were not relevant in terms of control of the disease and may not justify the application of differential sanitary strategies in these provinces.

**Table 2:** Province-level number of samples, positive results and BB prevalence for cattle and farms, with their confidence interval

Province	Cattle			Farms		
	Total	Reactive	Prevalence (%)	Total	Positive	Prevalence (%)
La Pampa	3,513	83	2.4 (1.6–3.2)^a^	187	48	26.0 (20.0–31.3)^a^
San Luis	5,452	74	1.4 (0.9–1.8)^b^	264	41	15.5 (11.8–19.3)^b^
Total	8,965	157	1.8 (1.3–2.2)	451	89	19.7 (17.0–22.4)

Prevalence values with different letters mean statistic differences *(P < 0.05)*

^a^ is the prevalence for La Pampa and ^b^ is for San Luis

Compared with the SENASA 2005 survey results, no significant differences were observed in the proportion of reactive cattle (*P* > 0.05 for both provinces). However, at farm level the prevalence was higher in both provinces, differences being significant only for La Pampa (*P* < 0.05).

None of independent variables assessed here were significantly associated with the farm-level infection status, nor with the number of reactive cows per farm.

A significant global clustering (Simes *P < 0.05*) of positive herds was detected using the Cuzick and Edwards’ test. Clustering of infected herds was detected at *k* = 1 (T*k* = 26, E [T*k*] = 17.4, *P* < 0.05). This result is consistent with patterns of local disease transmission.

**Fig. 3:**
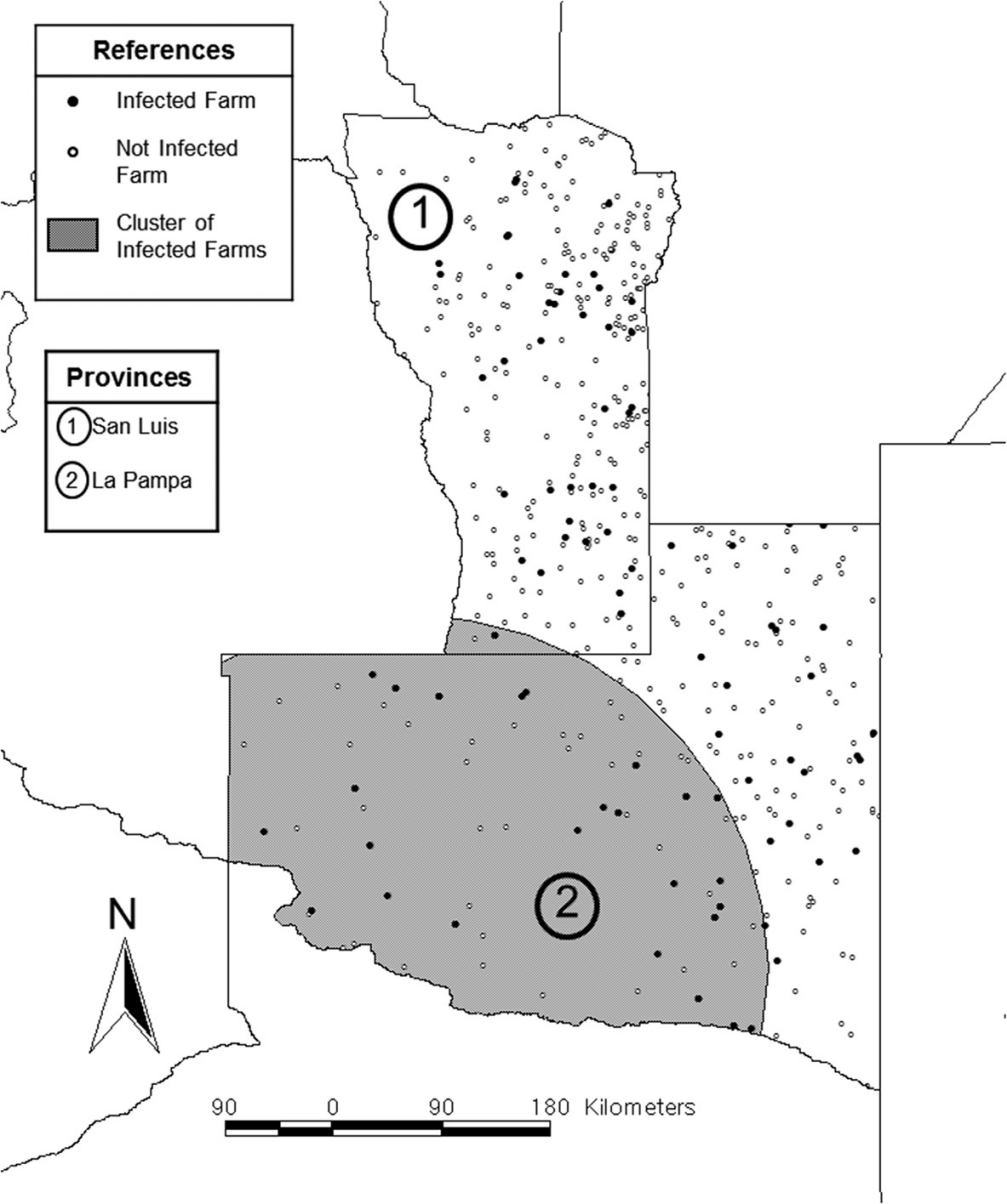
Geographic distribution of the uninfected (white dots) and infected (black dots) farms. The shaded area shows the cluster of infected farms detected by the Bernoulli model

Using the Bernoulli model, a spatial cluster of infected farms was detected in La Pampa (38.38 S, 66.92 W; radius = 250 km; *P* < 0.05) (Fig. 3), where the risk for infection was nearly 2.6 (RR = 2.58; *P* < 0.05) times higher in farms located inside the cluster than in those located elsewhere.

Contrarily, the Normal model was not significant, suggesting the absence of clusters that included farms with high within-farm prevalence.

The multivariate analysis showed that lower bovine densities and lower calf/cow ratio are associated with higher odds of being inside a disease cluster (*P* < 0.05), regardless the cut-off level used for categorization. Farm surface was significantly associated only when modeled as categorical variable using as cut-off value the median of the sampled farms (Table 3). Therefore, this variable was not included in the final model, which only includes bovine density and calf/cow ratio.

**Table 3:** Summary of logistic regression model showing the variables with significant association with the odds of being inside the cluster. Results are presented for both cut-off values used for independent variables

Variables	Cut-off (A)	OR (95 % CI)	Cut-off (B)	OR (95 % CI)
Surface	<300 ha	5.34 (0.72–39.69)	<500	12.47 (1.65–94.34)
Bovine density	≥0.24 animals/ha	8.23 (4.19–16.19)	≥0.5 animals/ha	4.99 (1.89–3.17)
Calf/cow index	≥0.36 animals/ha	2.63 (1.45–4.77)	≥0.5 animals/ha	3.31 (1.81–6.04)

## Conclusions

The increase of farm prevalence in the last five years suggests that the disease is spreading and that control measures should be applied in the region.

The cluster of infected farms was located in the west region of La Pampa described. There, farms have lower animal densities and smaller cow/calf indices compared to the rest of the province. Although no significant association was found between the risk of being in the cluster and farm surface when using both cut-off values for this variable (Table 3), results suggest an increased risk for being in the cluster in farms larger than 500 has. Those farms are managed more extensively and thus they are less productive, which likely correlates with infrequent veterinary control and poorer sanitary conditions of the herds, compared with smaller farms. Although western La Pampa has more infected herds, within-farm prevalence was not higher. This finding suggests that the control program has been relatively successful in controlling the disease at the farm level, and/or that low animal density inherently results in low disease prevalence. In any case, it is probable that within-farm prevalence was not sufficiently reduced so as to reduce the number of infected farms in the region.

Our results provide baseline information on the epidemiology of BB and its potential pattern of transmission in Argentina, which will ultimately help to improve BB control programs in the country.

## Abbreviations

BB: Bovine brucellosis
BPAT: Buffered plate agglutination test
CI: Confidence interval
DIC: Deviance information criterion
GLM: Generalized liner model
OR: Odds ratio
RR: Relative risk
SAT: Serum agglutination test
SENASA: National Service for Agrifood Health and Quality
SIGSA: Sistema Integrado de Gestión de Sanidad Animal
2ME: 2-mercaproethanol
